# Evaluation of a new Transmural Trauma Care Model (TTCM) for the rehabilitation of trauma patients: a study protocol

**DOI:** 10.1186/s12913-017-2037-2

**Published:** 2017-01-31

**Authors:** Suzanne H. Wiertsema, Johanna M. van Dongen, Edwin Geleijn, Maaike Schothorst, Frank W. Bloemers, Vincent de Groot, Raymond W. J. G. Ostelo

**Affiliations:** 1 Department of Rehabilitation Medicine, VU University Medical Center and the EMGO institute for Health and Care Research, PO BOX 7057, 1007 MB Amsterdam, The Netherlands; 2 Department of Health Sciences, VU University and the EMGO institute for Health and Care Research, Amsterdam, The Netherlands; 3 Department of Rehabilitation Medicine, VU University Medical Center, Amsterdam, The Netherlands; 4 Department of Trauma Surgery, VU University Medical Center, Amsterdam, The Netherlands; 5 Department of Epidemiology and Biostatistics, VU University Medical Center and Department of Health Sciences VU University and the EMGO institute for Health and Care Research, Amsterdam, The Netherlands

**Keywords:** Trauma, Fractures, Rehabilitation, Transmural care, Cost-effectiveness

## Abstract

**Background:**

Improved organization of trauma care in the acute phase has reduced mortality of trauma patients. However, there has been limited attention for the optimal organization of post-clinical rehabilitation of trauma patients. Therefore we developed a Transmural Trauma Care Model (TTCM). This TTCM consists of four equally important components: 1) intake and follow up consultations by a multidisciplinary team consisting of trauma surgeon and hospital based trauma physical therapist, 2) coordination and individual goal setting for each patient by this team, 3) primary care physical therapy by specialized physical therapists organized in a network and 4) E-health support for transmural communication and treatment according to protocols. The aim of the current study is to assess the cost-effectiveness of the TTCM.

**Methods:**

Patients will be recruited from the outpatient clinic for trauma patients of the VU University Medical Center (VUmc) if they have at least one fracture and were discharged home. A *controlled-before-and-after* study design will be used to compare the TTCM with regular care. Measurements will take place after the first outpatient clinical visit and after 3, 6 and 9 months. Prior to the implementation of the TTCM, 200 patients (50 patients per time point) will be included in the control group. After implementation 100 patients will be included in the intervention group and prospectively followed. Between-group comparisons will be made separately for each time point. In addition, the recovery pattern of patients in the intervention group will be studied using longitudinal data analysis methods. Effectiveness will be evaluated in terms of health-related quality of life (HR-QOL), pain, functional status, patient satisfaction, and perceived recovery. Cost-effectiveness will be assessed from a societal perspective, meaning that all costs related to the TTCM will be taken into account including intervention, health care, absenteeism, presenteeism and unpaid productivity. Additionally, a process evaluation will be performed to explore the extent to which the TTCM was implemented as intended, and to identify possible facilitators and barriers associated with its implementation.

**Discussion:**

This planned research will give insight into the feasibility of the TTCM model in clinical practice and will give a first indication of the cost-effectiveness of the TTCM and help us to further develop post-clinical trauma care.

**Trial registration:**

Trial registration number: NTR5474. The Netherlands National Trial Register (NTR). Registered 12 October 2015.

## Background

Trauma accounts for 9.6% of global mortality and is the leading cause of death during the first four decades of life [1, 2]. Since trauma patients are typically relatively young, the amount of Disability-Adjusted Life Years (DALYs) lost due to trauma, is larger than from any other disease and causes an important part of worldwide morbidity [3]. Furthermore, major trauma has shown to be the most important cause of long-term functional limitations in adults aged younger than 45 years [4].

The majority of trauma patients have one or more fractures due to their trauma, sometimes in combination with organ system injuries. Fractures of the lower extremities in particular have a major impact on functional status and health-related quality of life (HR-QOL) [5, 6]. Moreover, the economic burden of trauma to society is extensive due to the associated high *direct* as well as *indirect* costs (e.g. absenteeism costs). To illustrate, the total costs per patient with an operatively treated vertebral fracture is estimated to be €66.000, of which the majority (i.e. €47.000) is due to increased absenteeism [7]. Due to the major impact of trauma on mortality, morbidity, and (societal) costs, there has been increased interest in the organization of trauma care over the last three decades. In the literature it is frequently mentioned that trauma care is a chain of services, consisting of pre-hospital care, resuscitation and in-hospital care. During the last two decades, an improved organization of *pre-hospital* and *in-hospital* care by developing specialized trauma centers using Advanced Trauma Life Support (ATLS®) guidelines, has led to a 15–25% decrease in mortality of severe trauma patients [8–11]. Since mortality has decreased significantly due to this re-organization of trauma care, it has been suggested that the focus of trauma care should shift to *improving quality of life and outcome*, rather than on survival of trauma patients, because further improvements in survival rates are likely to be small [8, 11]. To improve quality of life and outcome among trauma patients, more attention for optimizing the rehabilitation phase is crucial. Even though numerous studies investigated the outcome of trauma patients, none of these studies focused on the *organization* and *content* of post-clinical trauma care. It is recognized that serious gaps exist between patients’ transition from acute care to rehabilitation and their return to society [12–14]. Therefore the limited focus on post-clinical trauma care is remarkable. Recently the American Trauma Society developed a post-clinical psychological support program, including self-management and peer support to improve the trauma patients’ psychosocial outcomes [15]. Nonetheless, there is limited attention for optimizing the organization of the post-clinical *physical* rehabilitation of trauma patients in primary care, which may have led to an inefficient and/or suboptimal rehabilitation process. After being discharged from a hospital, the majority of Dutch trauma patients rehabilitates in the primary care setting (i.e. treatment by a primary care physical therapist). In contrast to secondary and tertiary care, however, guidelines and protocols, as well as an interdisciplinary coordination, are lacking in primary care.

Previous research in other patient groups indicates that post-clinical care organized in networks of experienced and specialized healthcare providers is likely to result in better clinical outcomes and lower costs compared to regular care models [16]. Furthermore, a recent feasibility study among osteoarthritis patients showed improvements in health-related quality of life, function, and patient satisfaction when primary care was coordinated by a clinical case manager (mostly a hospital based physical therapist or nurse practitioner) who was in close contact with the surgeon [17]. However, whether such an organization of the post-clinical rehabilitation process of trauma patients also leads to improved treatment outcomes is currently unknown.

The aforementioned considerations led us to develop a new Transmural Trauma Care Model (TTCM) for trauma patients with at least one fracture, aiming to improve patient outcomes by refining the organization and quality of the post-clinical rehabilitation process. The TTCM is a joint initiative of hospital based physical therapists and trauma surgeons working closely together in the development of TTCM. The TTCM consists of four equally important components: 1) intake and follow up consultations by a multidisciplinary team consisting of a trauma surgeon and a highly specialized hospital based trauma physical therapist, 2) coordination and individual goal setting for each patient by this team, 3) primary care physical therapy by specifically trained trauma physical therapists organized in a network and 4) E-health support for transmural communication (between hospital based trauma physical therapist and primary care based physical therapist) and treatment according to protocols. To gain insight to the new care models’ cost-effectiveness a *controlled-before-and-after* study will be conducted [18]. This article describes the study protocol.

The proposed study aims to answer the following research questions:

1. Is the TTCM effective in terms of HR-QOL, pain, functional status, patient satisfaction and perceived recovery compared to regular care in trauma patients with at least one fracture?
2. Is the TTCM cost-effective from a societal perspective (including intervention costs, health care costs, absenteeism, presenteeism and unpaid productivity) compared to regular care?
3. What is the recovery pattern of patients receiving the TTCM in terms of HR-QOL, pain, functional status, patient satisfaction and perceived recovery during the 9 month follow-up period?
4. What are the barriers and facilitators associated with the implementation of the TTCM?
5. What is the reach, dose delivered, dose received, and fidelity of the TTCM?

## Methods

### Design

**Fig. 1:**
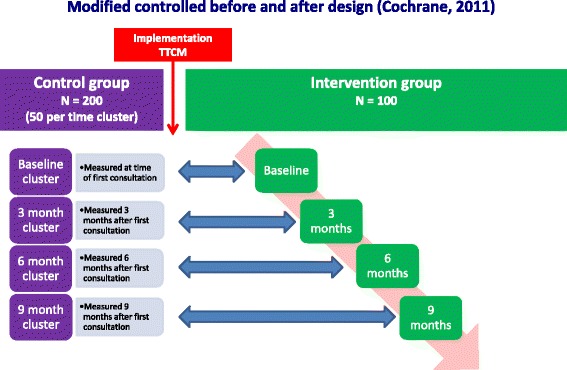
Study design

To answer the research questions, a modified *controlled-before-and-after* study will be conducted at the outpatient clinic for trauma patients of the VU University Medical Center (VUmc), Amsterdam, the Netherlands. The modification of the original study design -in which both control group and intervention group are observed prospectively- is that in our design only the intervention group will be prospectively followed. This modification is required due to the limited resources available. Prior to the implementation of the TTCM, data of 200 control patients who received care as usual will be collected during an inclusion period of 4 months. The control group will consist of 4 clusters of patients who either had their first consultation at the outpatient clinic for trauma patients of the VUmc 0 (i.e. baseline), 3, 6 or 9 months ago. Per cluster, we aim to include approximately 50 patients, all of whom will be asked to fill out an online questionnaire once after providing informed consent. After implementing the TTCM, patients who enter the outpatient clinic for trauma patients of the VUmc and meet the inclusion criteria will be asked to participate in the intervention group of the study. Patients in the intervention group will be prospectively followed for 9 months (*n* = 100) and will be asked to fill out online questionnaires at baseline, 3, 6 and 9 months after their first consultation at the outpatient clinic for trauma patients. See Fig. 1 for a detailed illustration of the study design.

### Population

A total of 300 trauma patients will be included in the study. Both operatively and non-operatively treated patients will be included, irrespective of whether or not they were admitted to the hospital. In order to be eligible for inclusion, trauma patients have to meet the following inclusion criteria: having at least one traumatic fracture, being aged >18 years, and being able to fill out online questionnaires. In both the intervention- and control group, the duration between the patients’ actual trauma and their first consultation at the outpatient clinic for trauma patients can vary, depending on the treatment that was selected at the emergency department (i.e. admitted to hospital or sent home). Patients will be excluded if they have red flags (i.e. traumatic brain injury, pathological fractures, and/or cognitive limitations), if they do not speak Dutch, if their rehabilitation process takes place in a tertiary care facility, and/or when patients live outside the catchment area of the VUmc.

### Recruitment

#### Control group

Control group patients will be identified from hospital records. All eligible patients will be contacted by phone by one of the investigators. At this point, patients receive further information about the study, and in- and exclusion criteria will be verified by the coordinating investigator. Patients who are willing to participate and eligible will then receive an email containing a link to an online questionnaire. Clicking the link to the online questionnaire will serve as informed consent. Patients who do not respond within 1 week will receive a reminder email which will be resent after another week of not responding. If the patient does not reply to both emails one of the coordinating investigators will contact the patient by phone to inquire whether the patient is still interested and willing to participate as indicated earlier.

#### Intervention group

Intervention group patients will be identified during their first consultation at the outpatient clinic for trauma patients. During this consultation, patients will be informed about the study purpose and procedures by one of the investigators. Also, in- and exclusion criteria will be verified. Patients who are willing to participate and are eligible will receive an email containing a link to an online questionnaire. Clicking the link to the online questionnaire will serve as informed consent. Subsequently, patients will be prospectively followed and will receive additional online questionnaires at 3, 6 and 9 months follow-up. Patients who do not respond within 1 week to one of the aforementioned online questionnaires will receive a reminder email which will be resent after another week of non-responding. If the patient does not reply to both emails one of the coordinating investigators will contact the patient by phone to inquire whether the patient is still interested and willing to participate.

### Intervention conditions

#### Regular care

Patients in the control group received regular care (i.e. trauma care that was provided at the VUmc prior to implementation of the TTCM). During regular care, the trauma surgeon acts as the chief consultant and performs the post-clinical consultations, unaccompanied by professionals of other disciplines. Based on personal judgement, the trauma surgeon decides if and when physical therapy in primary care is needed. After referral to a physical therapist, patients select a primary care physical therapist themselves, usually in their residential area. Moreover, during a patients’ treatment by a primary care physical therapist, there is typically no regular contact between the surgeon and the primary care physical therapist.

#### The transmural trauma care model (TTCM)

**Fig. 2:**
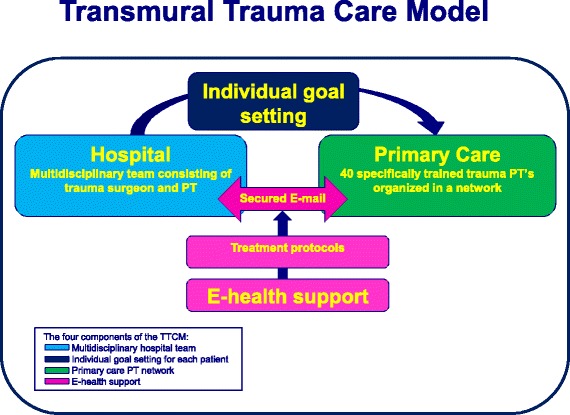
Schematic representation of the TTCM

Patients in the intervention group will receive care according to the TTCM at the outpatient clinic for trauma patients at the VUmc. Pre- and in-hospital trauma care remains unchanged and is equal to that provided to the control group. The essence of the TTCM is a regular feedback loop, in which the hospital team guides the primary care team by individual goal setting for each patient (see Fig. 2 for a schematic representation of the TTCM). The TTCM consists of four main components and will be explained below:

- *Intake and follow up consultations by a multidisciplinary team consisting of a trauma surgeon and a highly specialized hospital based trauma physical therapist.* The trauma surgeon acts as the chief consultant and is responsible for assessing the bone- and wound healing process and additional medical procedures, such as the prescription of medication and indicating surgery. The hospital based physical therapist, on the other hand, assesses physical function (e.g. mobility, strength, walking pattern). The trauma surgeon and hospital based physical therapist indicate -as a team- if and when physical therapy in primary care is needed.
- *Coordination and individual goal setting for each patient by the multidisciplinary hospital team.* This hospital team coordinates the patients’ rehabilitation process. The hospital based trauma physical therapist acts as case manager and repeatedly sets individual goals with the patient during the rehabilitation period.
- *Primary care physical therapy by specifically trained trauma physical therapists organized in a network.*
  This innovative “VUmc trauma rehabilitation network” consists of 40 physical therapists covering the region of Amsterdam. Patients in the intervention group with an indication for physical therapy treatment in primary care will be referred to one of the specialized trauma physical therapists of the VUmc trauma rehabilitation network. Prior to the implementation of TTCM, all 40 network physical therapists will follow a two-day training course led by trauma surgeons and hospital physical therapists. The course covers topics such as fracture healing, fracture treatment, complications and the most important principles of trauma rehabilitation. In addition, written working agreements will be discussed during the training course to assure optimal communication and use of IT services.
- *E-health support for transmural communication (between hospital based trauma physical therapist and primary care based physical therapist) and treatment according to protocols.*

For the purpose of the TTCM, an existing electronic patient record is adapted and ten rehabilitation protocols have been developed for the most common fractures (e.g. hip, tibia, ankle, proximal humerus, vertebra), which will function as guidelines for the primary care trauma physical therapists. The protocols are linked to a secured email device through which hospital physical therapist and the primary care physical therapist will communicate repeatedly throughout the whole rehabilitation process.

### Outcome assessment

An overview of all outcome measurements is provided in Table 1.

**Table 1:** Overview of all outcome measurements

Outcome	Measurement Instrument	Short term	Items	Itemscore	Interpretation
General ﻿HR-QOL	EQ-5D	EQ-5D	5	1–3	higher score: better health
Pain	Numeric Pain Rating Scale	NPRS	1	0–10	higher score: more pain
Perceived recovery	Global Perceived Effect	GPE	2	1–7	higher score: less recovery
Functional status	Patient Specific Function Scale	PSFS	3	100 mm VAS	higher score: less function
Patient satisfaction	Numeric Rating Scale	NRS	5	0–10	higher score: more satisfaction
Disease specific HR-QOL (upper extremity)	Quick Dash score	Q-DASH	11	1–5	sumscore 0–100, higher score: less function
Disease specific HR-QOL (lower extremity)	Lower Extremity Functional Scale	LEFS	20	0–4	sumscore 0–80, higher score: better function
Disease specific HR-QOL (vertebral fractures)	Roland Morris Disability Score	RMDS	24	yes/no	sumscore 0–24, higher score: more disability
Disease specific HR-QOL (multi trauma patients)	Groningen Activity Restriction Scale	GARS	18	1–4	sumscore 18–72, higher score: more restrictions
Healthcare utilization	Retrospective Cost Questionnaires				costs in Euro’s
Absenteeism	PROductivity and DISease Questionnaire	PRODISQ	1		total number of sick leave days
Presenteeism	WHO Health and Work Performance Questionnaire (NRS)	WHO-HPQ	1	0–10	higher score: better performance at work
Unpaid productivity loss			1		hours per week unable to perform unpaid activities

#### Baseline characteristics

At baseline, various demographic and trauma-related characteristics will be collected for all patients in the control- and intervention group, including age (years), gender (male/female), level of education (low/middle/high), medical history (none/chronic illness/musculoskeletal disease), type of trauma (traffic/fall/sport), injuries (upper extremity fracture/lower extremity fracture/vertebral fracture/multi trauma), treatment (operatively/conservatively), length of stay (days), and the well validated Injury Severity Score (ISS), used to provide an overall injury severity score for trauma patients [19]. The ISS score takes values from 0 to 75, and patients with an ISS > 16 are defined as multi-trauma patients. In the current study multi trauma patients are defined as having an ISS > 16 and/or having at least fractures in two or more extremities. Baseline characteristics will be collected using online questionnaires as well as data derived from electronic patient records.

#### Primary outcome measure

The primary outcome measure is general HR-QOL, measured using the Dutch version of the EQ-5D [20]. The EQ-5D consists of five questions representing five dimensions; mobility, self-care, usual activities, pain/discomfort and anxiety/depression. Each question is scored on a 3 point scale (1–3) with higher scores indicating greater severity level, resulting in a 5 digit index representing one of the 243 health status of the EQ-5D. Using the Dutch tariff, the participants’ EQ-5D health status will be converted into a utility score ranging from 0 (dead) to 1 (healthy). Additionally, quality adjusted life years (QALYs) will be calculated using linear interpolation between measurement points. The EQ-5D shows good psychometric properties in trauma patients with one or more fractures [21–23].

#### Secondary outcome measures

Secondary outcome measures include pain, perceived recovery, functional status, patient satisfaction, and disease-specific HR-QOL.

*Pain* will be measured using an 11-point numeric rating scale (NRS) ranging from 0 (no pain) to 10 (worst possible pain) [24]. Patients will be asked to rate their average pain over the last 7 days.

*Perceived recovery* will be measured using the Global Perceived Effect Scale. In clinical practice, measurement of patient-rated recovery often takes the form of the question: to what extent have you improved (or deteriorated) since last time? This type of rating of perceived recovery is a “transition scale” or Global Perceived Effect (GPE) scale, which has been advocated to increase the relevance of information from clinical trials to clinical practice [25]. From the patients’ perspective, the question is intuitively easy to understand and it allows them to rate those aspects of recovery that are most important to them. In the current study, patients will be asked the following question: “to what extend have you recovered since your trauma?” The GPE scale asks the patient to rate, on a 7 item scale, how much their condition has improved or deteriorated since their trauma. Possible answers include 1) completely recovered, 2) much improved, 3) slightly improved, 4) not changed, 5) slightly worsened 6) much worsened and 7) worse than ever [26].

*Functional status* will be measured using the Patient Specific Function Scale (PSFS), a patient specific outcome measure that is intended to complement the findings of generic- or condition-specific measures [27]. Patients will be asked to identify three important activities that they are having difficulty with or are unable to perform. Subsequently, patients are asked to rate their current level of difficulty associated with each activity, on an 11-point numeric rating scale ranging from 0 (“unable to perform activity”) to 10 (“able to perform activity at same level as before injury or problem”). The PSFS is translated and validated for the Dutch population [28].

*Patient satisfaction* will be scored using an 11 point numeric rating scale ranging from 0 (very dissatisfied) to 10 (excellent). Five patient satisfaction components related to the TTCM will be evaluated: 1) total treatment, 2) treatment at the outpatient clinic, 3) treatment in primary care, 4) collaboration between practitioners from the hospital team and 5) collaboration between the hospital team and the primary care physical therapist.

*Disease-specific HR-QOL* will be measured using one of the following disease-specific function scales, appropriate to the patients’ specific injury type (i.e. upper extremity fractures, lower extremity- and hip fractures, vertebral fractures and multi trauma patients):

Patients with fractures of the upper extremity will fill out the Quick Dash score, a short version of the Dash score (Disabilities of the Arm, Shoulder and Hand score) [29]. The Quick Dash score consists of 11 items, measuring physical function and symptoms on a five point scale (1–5 with higher scores indicating greater difficulty) in people with any or multiple musculoskeletal disorders of the upper limb. A validated Dutch version is available and will be used in this study [30].

Physical function in patients with hip fractures or other lower extremity fractures will be measured using the Lower Extremity Functional Scale (LEFS) [31]. The LEFS is a 20-item disease-specific questionnaire developed for measuring physical function in patients with musculoskeletal problems of the lower extremities. Each item is rated on a five point scale (0–4 with higher scores representing higher levels of functioning). The LEFS is frequently used as outcome measure in patients with fractures of the lower extremity [32]. A validated Dutch version of the LEFS will be used in this study [33].

Patients with vertebral fractures will fill out the Roland Morris Disability Score (RMDS) [34]. The RMDS is a disease-specific self-reported questionnaire consisting of 24 items all of which contain two answering categories (yes/no). The RMDS was originally developed for measuring function in patients with chronic low back pain, but is frequently used to evaluate outcome in patients with traumatic vertebral fractures (operated as well as conservatively treated). A validated Dutch version is available and will be used in this study [35].

Physical functioning in multi trauma patients will be assessed using the Groningen Activity Restriction Scale (GARS) [36]. The GARS is an 18 item scale on daily activities, all of which contain four response categories ranging from 1 to 4 representing 1 (being fully independent of other people) to four (being fully dependent of other people). The sum score provides information on the level of difficulty a person experiences in care taking and household activities. Recent research indicates good psychometric properties in a Dutch population of multi trauma patients [37].

#### Costs

Costs will be considered from a societal perspective, meaning that all costs related to the TTCM will be taken into account including intervention, health care, absenteeism, presenteeism and unpaid productivity. Except for intervention costs, costs will be assessed using retrospective cost questionnaires at baseline, 3, 6 and 9 months follow-up. Recall periods of these questionnaires will vary between treatment groups and measurement points in order to cover the complete duration of follow-up. To illustrate, 3-month recall periods will be used for the intervention group at all measurement points, whereas recall periods of 3, 6 and 9 months will be used for baseline/3-month follow-up, 6-month follow-up, and 9-month follow-up for the control group, respectively. All costs will be converted to the same reference year using consumer price indices. Discounting of costs will not be necessary due to the 9-month follow-up period.

*Intervention costs* will consist of all costs related to development and implementation of the TTCM (i.e. personnel costs, material costs, costs of the electronic patient record, educational costs). Intervention costs will be estimated using a bottom-up micro costing approach in which detailed data are collected regarding the TTCM’s units of resource use as well as their respective unit prices [38, 39].

*Health care utilization* will include primary care (e.g. consultations at the general practitioner or physical therapist) and secondary care (e.g. consultations at the outpatient clinic for trauma patients, hospitalization) as well as the use of medication. Dutch standard costs will be used to value health care costs [39]. Use of medication will be valued using the G-standard of the Dutch Society of Pharmacy [40].

*Absenteeism* will be retrospectively assessed using the “PROductivity and DISease Questionnaire” (PRODISQ) asking patients to report their total number of sick leave days [41]. Absenteeism will be valued using age- and gender-specific price weights [39].

*Presenteeism* is defined as reduced productivity while at work and will be assessed using the World Health Organization Health and Work Performance Questionnaire [42]. Presenteeism will be valued using age- and gender-specific price weights [39].

*Unpaid productivity losses* will be assessed by asking patients for how many hours per week they were unable to perform their unpaid activities, such as domestic work, school and voluntary work. Dutch shadow prices will be used to value unpaid productivity [39].

#### Process evaluation

A process evaluation will be performed to explore the extent to which the TTCM was implemented as intended as well as the possible facilitators and barriers associated with its implementation.

The extent to which the TTCM was implemented will be explored by assessing the four process evaluation components of Linnan and Steckler, including reach, dose delivered, dose received, and fidelity [43]. *Reach* is defined as the proportion of the intended target audience that eventually participated in the intervention (i.e. the TTCM). *Dose delivered* is defined as the number of intended units of the intervention provided (e.g. number of scheduled consultations/treatment sessions). *Dose received* is the extent to which trauma patients actively engaged in the intervention (e.g. number of attended consultations/treatment sessions in relation to the number that was scheduled). *Fidelity* is the extent to which the intervention was delivered as planned (i.e. the extent to which the intervention protocol was followed by the various care providers). To explore these four process evaluation components, data will be collected from the intervention group participants’ electronic patient records (e.g. number of secured emails, number of treatments in primary care, was the treatment according the protocol?).

Barriers and facilitators are defined as factors that hampered or enhanced the implementation of the TTCM, respectively [44]. For exploring the barriers and facilitators associated with the implementation of the TTCM, focus groups will be conducted among trauma patients (two focus groups consisting of five patients each), trauma surgeons (one focus group of six trauma surgeons), hospital based physical therapists (one focus group of five hospital based physical therapists), and primary care network physical therapists (two focus groups consisting of five primary care network physical therapists). Focus groups will be conducted at a time and location convenient to the participants. Prior to the focus groups, participants will be assured of confidentiality and will be asked to provide informed consent. The focus groups will be guided by two researchers, familiar with the TTCM, but not involved as care provider in the TTCM. During the focus groups, three round table discussions will be held; the first will be aimed at identifying possible facilitators, the second will be aimed at identifying possible barriers and the third round will be aimed at complementing and validating the barriers and facilitators identified in round one and two. During all round table discussions, a topic list will be used as a guide, but participants are allowed to discuss other topics that they consider to be of importance as well. All focus groups will be audiotaped and transcribed verbatim.

### Data analysis

#### Descriptive statistics

Descriptive statistics will be used to compare baseline characteristics between control- and intervention group participants and participants with complete and incomplete data.

#### Handling missing data

Missing data are assumed to be at random and will be imputed using Fully Conditional Specification and Predictive Mean Matching [45]. An imputation model will be constructed, including variables related to the “missingness” of data, variables that predict the outcomes, and all available midpoint and follow-up cost and effect measure values. The number of imputed data sets will be determined based on the number of participants with complete cost and effect measure values [46]. All of the imputed datasets will be analysed separately as specified below. Pooled estimates were subsequently calculated using Rubin’s rules [46].

#### Clinical effectiveness

The clinical effectiveness analyses will consist of two parts. First, the TTCMs’ effectiveness in terms of HR-QOL, pain, perceived recovery, functional status and patient satisfaction compared with usual care will be explored at 3, 6 and 9 months follow-up using regression analyses. The four clusters of control patients (i.e. time after their first consultation at the outpatient clinic for trauma patients respectively 0, 3, 6 and 9 months) will be compared with the patients in the intervention group at the corresponding time points. Second, the recovery pattern of patients in of the intervention group will be studied using longitudinal data analysis in terms of HR-QOL, pain, perceived recovery, functional status and patient satisfaction during the 9 month follow-up period (and while receiving the TTCM). All of the aforementioned analyses will be corrected for confounders if necessary (e.g. age, gender, level of education). Confounding will be checked by adding the potential confounding variable to the crude models, and will subsequently be considered to be present if the regression coefficient changes by 10% or more. All of the clinical effectiveness analyses will be performed in SPSS, using a level of significance of *p* < 0.05.

#### Economic evaluation

The economic evaluation will be performed from the societal perspective, meaning that all costs and consequences related to the intervention will be taken into account, irrespective of who pays or benefits. The mean difference in total costs between the intervention and control group will be compared to the corresponding mean difference in effects. For this, cost and effect differences will be estimated using seemingly unrelated regression analyses in order to correct for their possible correlation. To deal with the highly skewed nature of cost data, 95%CIs around the differences in costs will be estimated using the Bias Corrected and Accelerated Bootstrap method, with 5000 replications. Incremental Cost Effectiveness Ratios (ICERs) will be calculated by dividing the differences in costs by those in effects. To graphically illustrate the uncertainty surrounding the ICERs, bootstrapped incremental cost-effect pairs will be plotted on cost-effectiveness planes [47]. A summary measure of the joint uncertainty of costs and effects will be presented using cost-effectiveness acceptability curves, indicating the probability of an intervention being cost-effective in comparison with the control condition for a range of willingness-to-pay values (i.e. the maximum amount of money decision-makers are willing to pay per unit of effect gained) [48]. To test the robustness of the results, various sensitivity analyses will be performed [49]. All of the economic evaluation analyses will be performed in STATA, using a level of significance of *p* < 0.05.

#### Process evaluation

Using Nvivo, data derived from the focus groups will be analyzed in accordance to the constant comparative approach. That is, analytic categories will be inductively established by constantly comparing and checking items with the rest of the data [50]. By starting with open coding, descriptive themes and subthemes will be generated by one researcher. The final codes will subsequently be developed through discussion between two independent researchers. During these discussions, similar codes will be grouped into analytical categories and the different properties of these categories will be explored as well as the relationships between them (i.e. selective coding) [51]. Using SPSS, summary statistics will be prepared to evaluate the new care model’s reach, dose delivered, dose received, and fidelity.

## Discussion

Traumatic fractures are common and pose a substantial economic burden to society. Nonetheless, little is currently known about how to optimally organize the post-clinical rehabilitation process for trauma patients transferred from hospital to primary care. Therefore, the TTCM for the post-clinical rehabilitation of trauma patients was developed at the VUmc, which aims to improve HR-QOL, functional outcome and patient satisfaction of trauma patients, by organizing the post-clinical rehabilitation in an innovative and more efficient way. Within the available resources, the aforementioned *modified controlled before and after design* was regarded as the most optimal research design at this stage. The study aims to provide insight into the new care models’ cost-effectiveness and aims to provide clues as to how to further optimize the TTCM so it is “ready-to-implement” in other hospitals, which can possibly serve as a starting point for a future pragmatic (multicenter) controlled randomized trial. We are of the opinion that even though the applied *modified controlled before and after* design might bear on the internal validity of the current study findings (e.g. due to selection bias), it does not negate the value of its results. Another possible limitation of the proposed study might be the difficulty to identify what components of the TTCM will be responsible for (positive) effects. To illustrate, better clinical outcome could be the result of better educated physical therapists in primary care, but could also be due to the introduction of multidisciplinary consultations at the outpatient clinic for trauma patients. In the current study, a pragmatic design will be applied, in which the TTCM is evaluated as whole. Future research will therefore be needed to provide insight into which TTCM component is accountable for which specific effect.

Despite the shortcomings of the study we aim to provide insight in organizing the post-clinical rehabilitation process for trauma patients in a more efficient way and consequently contribute to better clinical outcomes and reduced societal costs.

## Abbreviations

ATLS®: Advanced trauma life support
DALY: Disability-adjusted life years
GARS: Groningen activity restriction scale
GPE: Global perceived effect
HR-QOL: Health-related quality of life
ICER: Incremental cost effectiveness ratios
ISS: Injury severity score
LEFS: Lower extremity functional scale
NRS: Numeric rating scale
NTR: The Netherlands National Trial Register
PRODISQ: PROductivity and DISease Questionnaire
PSFS: Patient specific function scale
RMDS: Roland Morris disability score
TTCM: Transmural trauma care model
VUmc: VU University Medical Center
